# Gender disparities in academic radiation oncology research: A narrative review

**DOI:** 10.1016/j.ctro.2026.101298

**Published:** 2026-09-27

**Authors:** Lucie Houdou, Angela Besserer, Maike Trommer, Audrey Larnaudie, Charles Raynaud, Véronique Vendrely, Stéphane Supiot, Yasmine El Houat, Anne Larrouy, Amandine Ruffier, Anne Laprie, Florence Huguet, Wassila Benhabib-Boukhelif

**Affiliations:** a Department of Radiation Oncology, Gustave Roussy, Villejuif, France; b Department of Radiation Oncology, Ernst von Bergmann Hospital Potsdam, Potsdam, Germany; c Department of Radiation Oncology, University Hospital Bonn, Bonn, Germany; d Department of Radiation Oncology, François Baclesse Centre, Caen, France; e Department of Radiation Oncology, Haut-Lévêque Hospital, Bordeaux, France; f Department of Radiation Oncology, Institut de Cancérologie de l'Ouest, Saint-Herblain, France; g Department of Radiation Oncology, Centre Hospitalier de Roanne, Roanne, France; h Department of Radiation Oncology, Paris-Nord Cancer Centre, Sarcelles, France; i Department of Radiation Oncology, Jean-Bernard Centre, Le Mans, France; j Department of Radiation Oncology, Oncopole, Toulouse, France; k Department of Radiation Oncology, Tenon Hospital, Paris, France; l Department of Radiation Oncology, Intercommunal Hospital Centre of Créteil, Créteil, France; m Assistance Publique - Hôpitaux de Paris (AP-HP), Paris, France

**Keywords:** Radiation oncology, Gender disparities, Academic medicine, Diversity, equity and inclusion, Women

## Abstract

**Introduction:**

Despite rising numbers of women in radiation oncology (RO), they remain under-represented in academic leadership and research. This narrative review assesses gender disparities in academic RO.

**Materials and methods:**

PubMed/MEDLINE and Europe PMC were searched in August 2026 for articles published from 2000 onwards, with additional domain-specific and country-specific searches. Of 1629 records, 1035 were screened on title and abstract after removal of duplicates and preprints, 657 were assessed in detail, and 108 that reported primary, gender-disaggregated data on radiation oncologists or trainees were included, together with three authorship analyses that report the radiation oncology journals separately. Because outcome definitions and academic structures differ across countries, the synthesis is narrative.

**Results:**

The “leaky pipeline” was a consistent finding: women hold a smaller share of senior posts than of the workforce in every country with data. Across United States National Cancer Institute-designated centres, 24.6% of men versus 15.5% of women held full professorships (*p* = 0.001), and women were 11.8% of chairs at those centres. Women held 18.9% of full professorships in Germany in 2024. Crude bibliometric differences were attenuated or abolished by stratification for rank. Men were 76% of 340 National Institutes of Health-funded RO principal investigators, and women were twice as likely as men to treat breast malignancies (risk ratio 2.01, 95% CI 1.75–2.50). In France, 65% of female respondents perceived gender as a career barrier. Coverage is uneven: 82 of 108 studies are North American, none from Africa.

**Conclusion:**

Disparities persist across countries. The evidence is observational and cannot establish mechanisms; corrective programmes report outcomes, none controlled. Differences are associations, not causal effects; no data were identified for Africa, the Middle East, Eastern Europe or most of Asia. A national survey with SFRO, SFjRO and FORce is needed.

## Highlights

• Gender disparities persist across academic radiation oncology worldwide.
• 108 studies from two databases; 82 are North American, none from Africa.
• Gaps affect rank, leadership, authorship, funding and disease-site allocation.
• Evidence is observational and largely unadjusted; mechanisms remain hypothetical.
• Corrective programmes report outcomes, but none is controlled.

## Abbreviations

**French academic-hospital system**
PU-PH: professeur des universités, praticien hospitalier (full professor and hospital practitioner)
MCU-PH: maître de conférences des universités, praticien hospitalier (associate professor and hospital practitioner)
CNU: Conseil National des Universités, which qualifies candidates for academic posts
CNG: Centre National de Gestion, which administers appointments to academic-hospital posts
HDR: Habilitation à Diriger des Recherches, the qualification required to supervise doctoral work
PHRC: Programme Hospitalier de Recherche Clinique, the principal national funders of clinical cancer research
INCa: Institut National du Cancer, the principal national funders of clinical cancer research
CNOM: Conseil National de l'Ordre des Médecins, the national medical register

**Professional societies and organisations**
SFRO: French Society for Radiation Oncology
SFjRO: its junior members' society
FORce: Femmes Oncologues Radiothérapeutes pour la coopération et l'équité, a French professional group for women in the specialty
ESTRO: European Society for Radiotherapy and Oncology
ASTRO: American Society for Radiation Oncology
ABS: American Brachytherapy Society
DEGRO: German Society of Radiation Oncology
FiRO: Frauen in der Radioonkologie, the Women in Radiation Oncology Working Group of DEGRO
CARO: Canadian Association of Radiation Oncology
RANZCR: Royal Australian and New Zealand College of Radiologists
SCAROP: Society of Chairs of Academic Radiation Oncology Programs
SWRO: Society for Women in Radiation Oncology.

**Other**
RO: radiation oncology
NCI: National Cancer Institute
NIH: National Institutes of Health
CI: confidence interval
RR: risk ratio
URM: under-represented in medicine

## 1 Introduction

In its 2030 vision of “Optimal Health for All, Together”, the European Society for Radiotherapy and Oncology (ESTRO) emphasises the urgent need to advance diversity, equity, and inclusion as drivers of both healthcare professionals' well-being and the delivery of high-quality care, research, and innovation [1].

Promoting gender diversity is not only a matter of representation and fairness but also a driver of scientific quality [2]. Female principal investigators enrol a higher proportion of women in clinical trials, design more inclusive protocols and lead gender-diverse teams [3], [4], [5], which matters where under-representation of women in studies has generated biased evidence, particularly in cancers prevalent among women [6]. Diversity within research teams is associated with broader innovation and higher research quality [7].

Over the past two decades the proportion of women enrolling in medical schools has reached or exceeded 50% in most Western nations, a trend increasingly reflected in radiation oncology (RO) [8], [9]. This numerical increase at entry does not yet translate into equitable representation in academic research and institutional leadership [10], [11].

In France, the proportion of female radiation oncologists has increased in recent years, a trend already documented among trainees in 2008 [12]. Women account for approximately 44% of practising radiation oncologists, a background figure quoted by the authors of the French national survey rather than a finding of that survey [13], yet their presence in senior academic positions is disproportionately low: in 2024 men held 74.4% of full professor-hospital practitioner (PU-PH) positions across all medical disciplines, against 80.8% in 2016 (Centre National de Gestion, Bilan Hospitalo-Universitaire, 2024).

This narrative review, based on a systematic search, brings together the radiation-oncology-specific evidence on gender disparities in academic careers, characterises the quality of that evidence, distinguishes documented disparities from reported experiences, proposed mechanisms and evaluated interventions, and identifies what remains unknown, particularly in France.

## 2 Materials and methods

### 2.1 Type of review

This is a narrative review based on a systematic search: the search, screening and eligibility criteria are reproducible and reported below, but the synthesis is narrative, because the literature cannot support a pooled synthesis. Seniority is defined differently in every national system, so a pooled proportion across the French PU-PH/MCU-PH structure, the German Habilitation track and the North American professorial ladder would not correspond to any real quantity. Denominators are drawn largely from overlapping national censuses and society rosters rather than from independent samples, so successive censuses of the same workforce are reported as a series rather than pooled. Bibliometric outcomes are defined incompatibly, one analysis using a career-wide h-index and another an index restricted to residency. Gender is also ascertained in four different ways across the evidence base (Section 3.8), so the exposure is not the same construct throughout. The narrative form has also allowed us to give due weight to national workforce censuses and society analyses that a pooled synthesis could not accommodate, and to broaden the geographical scope of the review.

### 2.2 Search strategy

A systematic search was conducted to identify studies of gender disparities in the academic and research workforce in radiation oncology. PubMed/MEDLINE was searched on 10 August 2026 and Europe PMC on 12 August 2026, in both cases for articles published from 1 January 2000 onwards, with no language restriction. The search was executed using a combination of Medical Subject Headings (MeSH) and free-text keywords, structured in three concept blocks: radiation oncology, gender, and the academic or professional workforce. The same three blocks were translated into the Europe PMC field syntax. The complete search string for each database, the date limits applied, and a note on two features of PubMed syntax that fail silently and that affected our initial search are given in **Appendix A.**

Both searches were supplemented by domain-specific searches and by country-specific searches for Australia and New Zealand, Japan, India and South Asia, Germany, France, Latin America, the Middle East and Africa, undertaken to counter the North American concentration of this literature. The bibliographies of retrieved articles were manually screened, and the websites of ESTRO, ASTRO, DEGRO, CARO, RANZCR and SFRO were checked for workforce reports. Embase, Scopus and Web of Science could not be searched, no institutional subscription to those databases being available to the authors.

### 2.3 Screening and study selection

PubMed/MEDLINE returned 749 records and Europe PMC 880, a total of 1629; removing the 565 records common to both left 1064. Twenty-nine preprints were set aside as not peer reviewed, leaving 1035 records whose title and abstract were read; the only relevant one is included here in its peer-reviewed form, so no evidence was lost. A first pass excluded 378 PubMed records lacking a title or abstract term relating simultaneously to gender, radiation oncology and the workforce, the Europe PMC field tags having imposed that constraint at search. In total 657 records were assessed in detail.

Inclusion criteria were: (1) original studies, including cross-sectional, registry, cohort, bibliometric, survey, qualitative and workforce-census designs, addressing gender in radiation oncology; (2) studies reporting on research productivity, academic rank, funding, leadership, recognition, disease-site allocation, compensation, workplace experience, parenthood, the training pipeline, or self-reported career barriers; (3) studies presenting primary, gender-disaggregated data specific to radiation oncologists, trainees or departments. Exclusion criteria included general oncology without radiation oncology data, editorials without primary data, and studies of clinical patient outcomes only.

Of these, 111 reports were retained for use: 108 met all three criteria and constitute the evidence base, and three are authorship analyses of oncology at large that report the radiation oncology journals separately and are therefore counted in addition to the 108, for the reason given below. Of the remaining 546 records, 515 failed the eligibility criteria, most often for a patient population, no primary or gender-disaggregated data, or another specialty with no radiation oncology group reported separately; eight of those 515 were conference abstracts of studies already included as full papers, and 15 were excluded only at a second audit of records initially retained, nine for a population that proved not to be radiation oncology or for absent gender-disaggregated results and six because they were adjacent-specialty reports with no separate radiation oncology group. The remaining 31 records concern academic medicine or oncology at large; they are eligible for use as context and are cited in Section 4.3 where relevant. Fig. 1 shows the flow of records and Fig. 2 the geographical distribution. Two exceptions to criterion (3) are identified at the point of use. The first admits an analysis of another specialty, or of oncology at large, that reports radiation oncology as an identified subgroup; three authorship analyses are included under it, and these are the three reports counted in Fig. 1 in addition to the 108. The second admits a single national report that is the only source for its region; that report is counted within the 108.

**Fig. 1:**
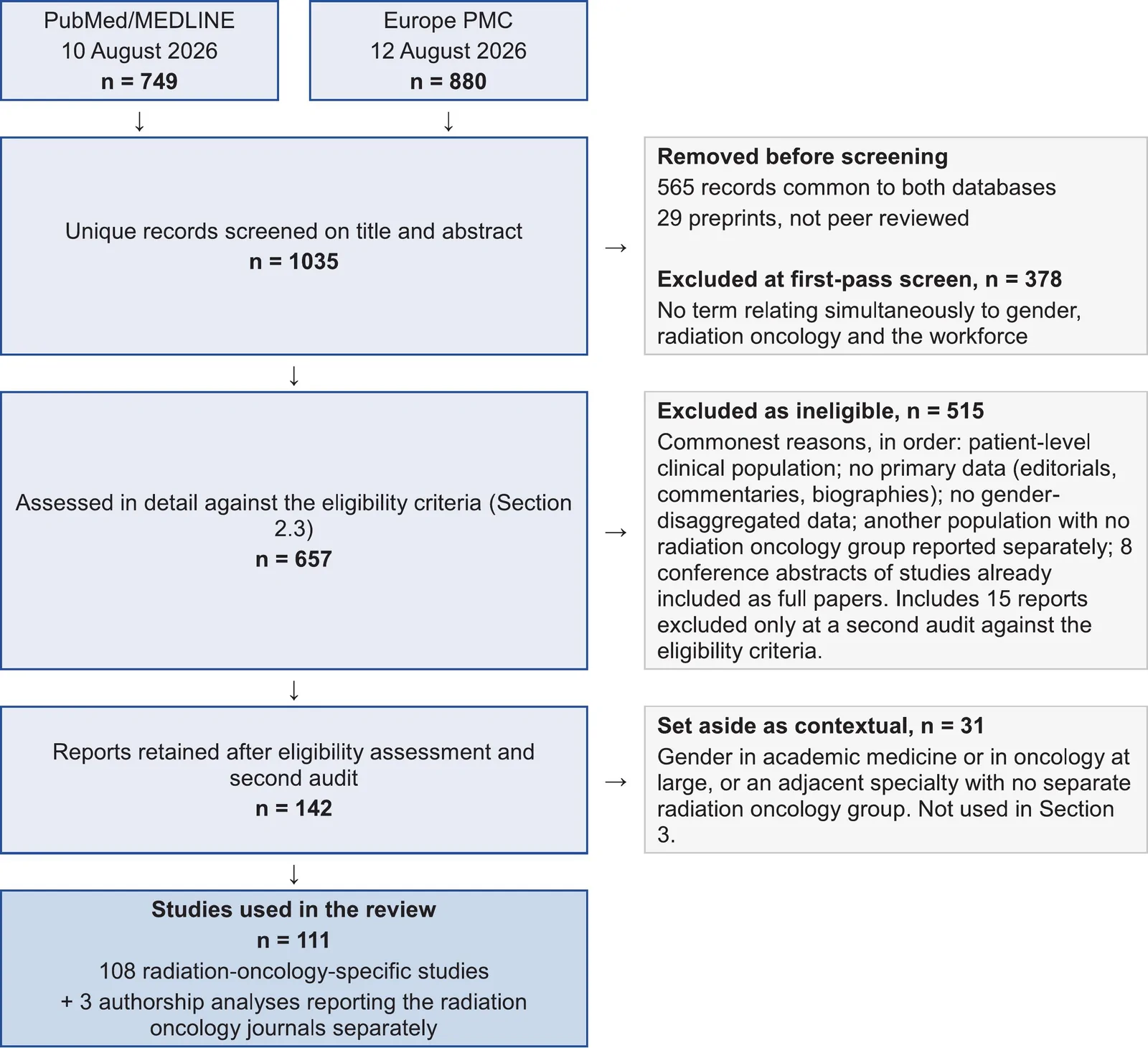
Flow of records through both searches and the selection process. **Legend:** This diagram records the yield of both searches and the reasons for exclusion. It is deliberately not presented as a PRISMA 2020 flow diagram: PRISMA 2020 is designed for systematic reviews, and the synthesis presented here is explicitly narrative. The diagram nonetheless reports every count a PRISMA 2020 flow diagram would contain. Every retained report was re-assessed in a second audit against the eligibility criteria; the 15 reports excluded at that stage are counted once, within the 515 ineligible records. The three retained authorship analyses report the radiation oncology journals separately and are counted in addition to the 108 radiation-oncology-specific studies.

**Fig. 2:**
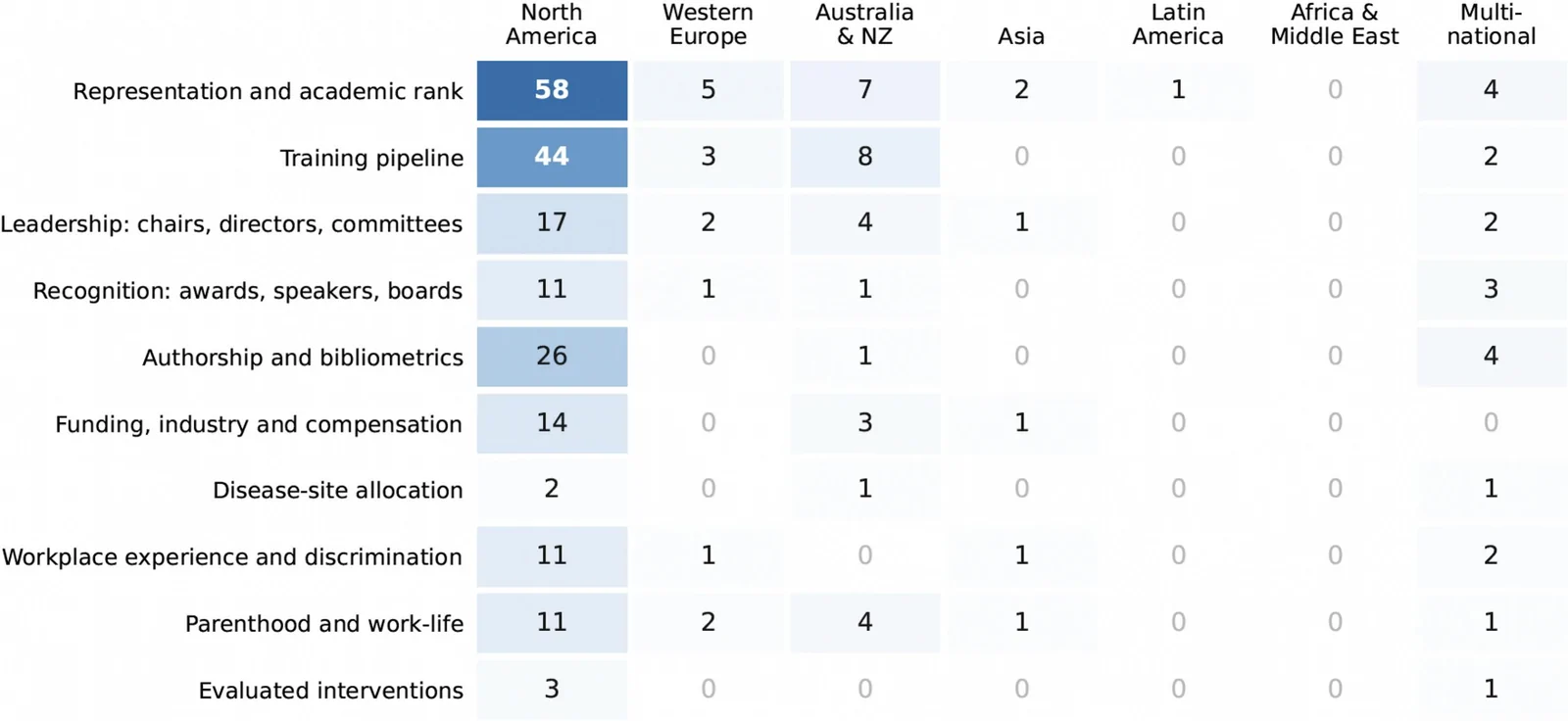
Geographical coverage of the radiation-oncology-specific evidence, by domain and region. **Legend:** Cell values are the number of the 108 included studies contributing gender-disaggregated data in that domain and region; a study contributing to several domains is counted once in each. The empty and near-empty cells are the substantive finding. We identified no radiation-oncology-specific study reporting gender-disaggregated data for Africa or the Middle East in any domain, and a single study for Latin America. Funding, compensation and disease-site allocation have been examined almost exclusively in the United States. Studies of oncology at large, and adjacent-specialty studies in which radiation oncology is a subgroup, are cited in the text where they are the only source available but are not counted here.

### 2.4 Sex and gender terminology

Throughout this review, sex refers to biological attributes and gender to socially constructed roles and identities. Because no study discussed distinguishes the two and effectively all use binary categories, we use gender and the categories women and men to reflect the source data, and report for each key study how gender was determined. Almost no report enumerates non-binary or gender-diverse practitioners; a recent Canadian survey collecting gender identity and sexual orientation is a welcome exception [14]. This absence is a property of the evidence base, not a choice of ours, and it leaves gender-diverse radiation oncologists largely invisible below.

### 2.5 Data extraction and appraisal

Screening and data extraction were performed by L.H. All studies retained for inclusion, and all data extracted into Table 1, Table 2, were independently checked against the source reports by a second author (W.B.), and disagreements were resolved by discussion. Records excluded at screening were not re-screened in duplicate, which Section 4.6 states as a limitation.

**Table 1:** Characteristics of the 108 radiation-oncology-specific studies included in the review.

Characteristic	Studies, n (%)
**Region**	
North America	82 (76%)
Western Europe	5 (5%)
Australia & NZ	10 (9%)
Asia	2 (2%)
Latin America	1 (1%)
Multinational	8 (7%)

**Year of publication**	
2000 to 2009	8 (7%)
2010 to 2019	40 (37%)
2020 to 2026	60 (56%)

**Principal design**	
Survey	45 (42%)
Bibliometric analysis	26 (24%)
Cross-sectional audit or database analysis	25 (23%)
Workforce census or registry analysis	7 (6%)
Cohort study	3 (3%)
Qualitative study	2 (2%)

**Method of gender ascertainment**	
Self-reported by participants	43 (40%)
Administrative or registry field	18 (17%)
Attributed by investigators from names, photographs or websites	27 (25%)
Method not stated	20 (19%)

**Evidence domain addressed**	
Representation and academic rank	77 (71%)
Training pipeline	57 (53%)
Authorship and bibliometrics	31 (29%)
Leadership	26 (24%)
Parenthood and work-life	19 (18%)
Funding, industry and compensation	18 (17%)
Recognition, awards and visibility	16 (15%)
Workplace experience and discrimination	15 (14%)
Disease-site allocation	4 (4%)
Evaluated interventions	4 (4%)

**Statistical reporting**	
Reports at least one *p* value	61 (56%)
Reports at least one adjusted effect estimate	17 (16%)
Reports at least one confidence interval	10 (9%)

**Legend:** Percentages are of the 108 included studies and do not sum to 100% where a study contributes to more than one category (for example in evidence domain addressed). Studies classified as multinational report data spanning more than one region, most often journal authorship or society membership. Three further reports that analyse authorship across several oncology disciplines and report the radiation oncology journals separately are cited in the text under the exception defined in Section 2.3 but are not included in this table, nor are the contextual sources used in Section 4.3.

**Table 2:** Effect estimates and their precision, for the included studies that report them.

Domain	Study [reference]	Population and outcome	Estimate **[**95% CI**]**	p	Adjusted
Representation and rank	Kamran 2022 [15]	US radiation oncology faculty, 1970 to 2019; decadal trend in the proportion who are non-URM women	+0.4% per decade [0.25 to 0.80]	<0.001	No (trend)
Representation and rank	McClelland 2022 [11]	993 faculty at 51 NCI-designated comprehensive cancer centres; holding full professor rank	24.6% of men vs 15.5% of women	0.001	No
Representation and rank	Raldow 2023 [16]	858 academic radiation oncologists from 63 US departments (2018 SCAROP Financial Survey); odds of holding associate or full professor rank, women vs men	OR 0.86 [0.56 to 1.32]	0.48	Yes
Representation and rank	Talom 2026 [17]	US academic radiation oncologists; odds of promotion, women vs men	OR 1.02 [0.68 to 1.52]	0.94	Yes
Leadership	Pourghadiri 2025 [18]	North American radiation oncology society committees; leadership positions held by men	73.5%	<0.0001	No
Leadership	Beeler 2019 [19]	US academic radiation oncology chairs; extramural grant funding held at first chair appointment, men vs women	median $1.89 million vs $0.25 million	0.006	No
Recognition	Huang 2021 [20]	Speaker introductions at the ASTRO annual meeting; odds of a professional form of address for a woman speaker	OR 0.36 [0.26 to 0.49]	<0.001	Yes
Recognition	Khullar 2024 [21]	ARRO Educator of the Year award; odds of a female awardee in a large versus small programme	OR 6.86 [2.71 to 23.1]	<0.001	No
Recognition	Besserer 2026 [22]	2795 panellists across 85 German oncological guidelines; women among the 255 radiation oncology panellists	34.5%	<0.05	No
Authorship and bibliometrics	Lee 2020 [23]	Abstract-presenter and senior-author dyads; odds of subsequent higher-impact publication for a female-male dyad	OR 2.48 [1.32 to 4.66]	not reported	Yes
Authorship and bibliometrics	Jones 2023 [24]	910 US residency graduates 2015 to 2019; first-author publications during residency, women vs men	adjusted beta −0.75	0.005	Yes
Authorship and bibliometrics	Choi 2009 [25]	826 US academic radiation oncologists; mean h-index, women vs men	6.4 vs 9.4	not reported	No
Authorship and bibliometrics	Anabaraonye 2022 [26]	Radiation oncology publications during the early COVID-19 period; non-senior female first authors of scientific manuscripts	37% vs 11%	0.02	No
Authorship and bibliometrics	Quinn 2024 [27]	168 RANZCR radiation oncology fellows completing training 2014 to 2023; number of first-author publications during training, by gender (any-author publications, *p* = 0.002)	not reported	0.04	No
Funding and compensation	Mutwiri 2022 [28]	340 NIH-funded radiation oncology principal investigators, 2016 to 2019; proportion who are men	76%	not reported	No
Funding and compensation	Weng 2019 [29]	US Open Payments database; median difference in consulting payments, women vs men	-$1000 [−1966.67 to 100.63]	0.005	No
Funding and compensation	Guss 2019 [30]	US public academic radiation oncology departments; mean salary difference, women vs men, unadjusted Mean salary difference after adjustment for rank and years in practice	−12.6% (−$48,974) −6.4% (−$26,458)	<0.01 <0.01	No Yes
Funding and compensation	Raldow 2023 [16]	858 academic radiation oncologists; total compensation, women vs men, adjusted	−1.7% [−6.8% to 3.4%]	0.51	Yes
Funding and compensation	Valle 2019 [31]	4393 US radiation oncologists, 2016 Medicare data; Medicare revenue collected, women vs men, non-facility-based practice Facility-based practice; Medicare charges submitted, women vs men	-$143,610 [−$185,528 to -$101,692] −423 charges [−506 to −341]	<0.001 <0.001	No No
Disease-site allocation	Corrigan 2024 [32]	1337 US academic faculty, January 2020; likelihood of treating breast cancer, women vs men Likelihood of treating gynaecological cancer, women vs men Likelihood of treating genitourinary cancer, women vs men	RR 2.01 [1.75 to 2.50] RR 2.06 [1.72 to 2.79] RR 0.40 [0.34 to 0.48]	<0.001 <0.001 <0.001	No No No
Workplace experience	Gasnier 2022 [1]	ESTRO membership survey, 812 respondents from 35 countries; favourable overall inclusion score, respondents reporting minority status vs not. The gender-minority subgroup scored 47%.	58% vs 71%	<0.001	No
Workplace experience	Allen 2025 [33]	98 Canadian radiation oncology trainees; perceived under-representation during training, women vs men Same population; perceived discrimination events, women vs men	46% vs 13% 64% vs 19%	0.001 <0.001	No No
Parenthood	Dover 2022 [34]	Early-career female radiation oncologists; infertility among those who intentionally delayed pregnancy vs not	46% vs 18%	0.008	No
Training pipeline	Mattes 2022 [35]	981 ASTRO members; women among student members vs members-in-training	40.0% vs 31.5%	0.032	No
Training pipeline	Mattes 2020 [36]	US medical schools; correlation between the number of female radiation oncology faculty and the percentage of graduates entering the specialty	*r* = 0.481	<0.001	No
Training pipeline	Dhami 2016 [37]	US radiation oncology residents; odds of satisfaction with mentorship in a formal mentoring programme	OR 5.0	<0.001	Yes

**Legend:** Estimates are reproduced as published, including the direction of the referent category; the bracketed number after each study is its reference-list number. Only 61 of the 108 included studies report a p value, 17 an adjusted estimate and 10 a confidence interval, so this table covers the minority of the evidence base for which precision can be assessed. Null results (Raldow 2023, Talom 2026) come from samples not powered for the stratified comparison and are failures to detect a difference, not demonstrations of equivalence; where adjustment is for academic rank, rank is itself an outcome under study. The Medicare estimates measure billing activity, not salary, and are unadjusted for clinical volume, case mix or contracted hours. Two intervals are internally unusual but verified against the published articles and reproduced unaltered: the Weng 2019 interval accompanies a rank-based p value and so does not share its estimator, and the upper bounds of the two positive Corrigan 2024 risk ratios are asymmetric on the log scale.

For each included study, we extracted the region, study period, sampling frame, sample size, gender-specific denominators, how gender was determined, the outcome definition, the effect estimate with its confidence interval and *p* value, whether the analysis was adjusted, and the response rate, where reported. Each study was appraised across five domains: sampling frame, gender-ascertainment validity, outcome measurement, confounding adjustment and completeness of data, with non-response bias added for surveys. No validated critical-appraisal instrument exists for bibliometric research, hence this domain-based framework. The appraisal is summarised in Table 1 and Section 3.8 and study by study in Table 2; no report was set aside on appraisal grounds, it being used to weight interpretation.

### 2.6 Synthesis of results and labelling of evidence

Given the heterogeneity of designs and metrics, described in Section 2.1, a qualitative synthesis was performed, organised by evidence domain. Evidence is labelled in three tiers, the label stated wherever a claim is made. Radiation-oncology evidence is primary, gender-disaggregated data on radiation oncologists, trainees or departments; Section 3 reports this and no other, apart from the two exceptions defined in Section 2.3. Contextual evidence comes from general academic medicine, radiology or oncology at large, is confined to Section 4.3 and is labelled there, with the limits of extrapolation stated. Interpretation and hypotheses are confined to Section 4.2 and identified there as untested within radiation oncology.

### 2.7 Ethical approval

Institutional review board approval was not required, as this study did not involve human participants and relied exclusively on publicly available data.

## 3 Results

### 3.1 Characteristics of the literature identified

One hundred and eight studies constitute the evidence base, and their distribution is uneven in both geography and subject matter, which is itself a finding (Fig. 2). Evidence is concentrated in the United States and Canada, with a smaller Western European body, a substantial Australasian one, isolated Asian reports and a single Latin American report; we identified no radiation-oncology-specific, gender-disaggregated study from Africa or the Middle East. Representation and academic rank (*n* = 77 studies) and the training pipeline (*n* = 57) are well covered; authorship and bibliometrics (*n* = 31), leadership (*n* = 26), parenthood and work-life (*n* = 19), funding, industry payments and compensation (*n* = 18), recognition (*n* = 16) and workplace experience (*n* = 15) less so; disease-site allocation (*n* = 4) and evaluated interventions (*n* = 4) rest on a handful of reports each. Gender ascertainment and reporting quality are set out in Section 3.8. Designs are almost entirely cross-sectional, registry-based, bibliometric or survey-based, so no causal inference is available. Table 1 summarises the evidence base, Table 2 gives the effect estimates that exist, and Fig. 3 displays the proportion of women across successive indicators. Table 3 summarises, for each evidence domain, the direction of the disparity reported, the strength of the underlying evidence and the principal gap that remains.

**Fig. 3:**
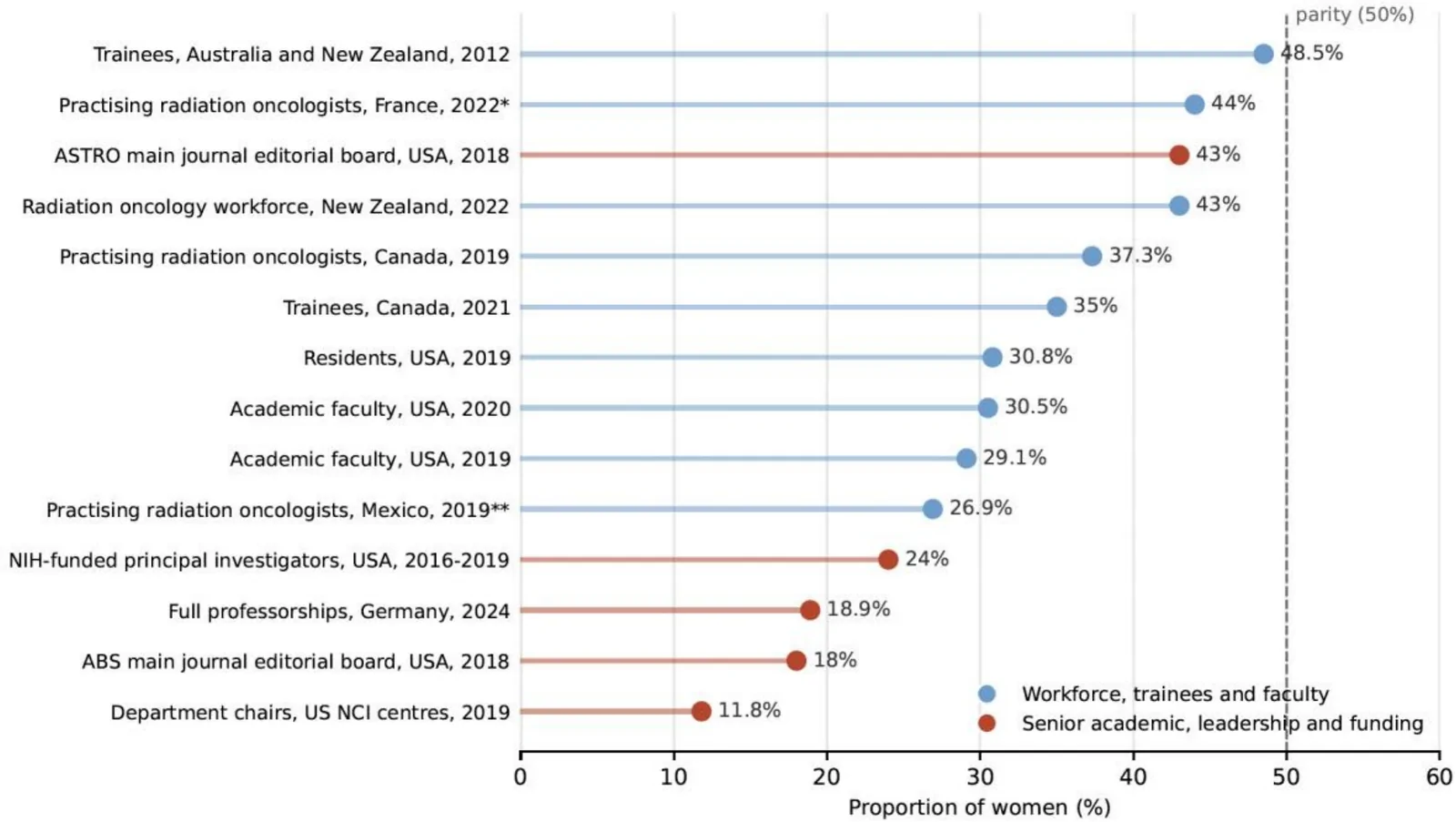
Proportion of women across successive indicators in academic radiation oncology. **Legend:** Each indicator is reported as its source publishes it, with the population, country and year stated. The indicators come from different countries, denominators and years and are displayed together for illustration, not as a single pooled series. **Abbreviations:** ABS, American Brachytherapy Society; ASTRO, American Society for Radiation Oncology; NCI, National Cancer Institute; NIH, National Institutes of Health. *Background figure quoted by the authors of the French national survey [13], not a finding of that survey. **Radiation oncologists certified by Mexico's Radiation Oncology Certification Board [38].

**Table 3:** Gender disparities in academic radiation oncology at a glance.

Domain	n	Direction	Key estimate	Evidence	Main gap
**Career progression and rank**	77	**↓ Fewer women at each senior step**	15.5% vs 24.6% full professors (US, *p* = 0.001)	**Moderate**	No longitudinal cohort; no French data
**Leadership and governance**	26	**↓ Chairs, committees, boards** **↑ Diversity and wellness roles**	11.7% of chairs vs 30% of faculty	**Limited**	Appointment processes; non-US data
**Recognition and visibility**	16	**↓ Invited lectures and honoraria**	27% of 725 invited ASTRO speakers	**Limited**	Eligible-pool denominators; non-US societies
**Productivity and authorship**	31	**↓ Crude output and senior authorship** **↔ After adjusting for rank**	h-index 6.4 vs 9.4; gap lost on stratification	**Moderate**	Career-duration-matched analyses
**Funding, PI roles and pay**	18	**↓ PI roles and industry payments** **↓** or **↔ Pay, by adjustment**	76% of 340 NIH-funded PIs are men	**Limited**	Application vs success rates; EU funders
**Disease-site allocation**	4	**↑ Breast and gynaecology** **↓ Genitourinary**	RR 2.01 for breast (1.75–2.50)	**Very limited**	Choice vs assignment; mediation untested
**Workplace and parenthood**	15/19	**↑ Bias, harassment, burnout** **↑ Leave and career effect of children**	62% of French women report harassment	**Limited**	Representative frames; male comparators
**Interventions**	4	**↔ No effect demonstrated**	No controlled evaluation	**Very limited**	Controlled evaluation, hard outcomes

**Legend**: Arrows give the direction reported for women relative to men: ↓ lower or under-represented, ↑ higher or over-represented, ↔ no difference detected. n is the number of included studies addressing the domain (Table 1); the total exceeds 108 because a study may address several domains. One estimate is given per domain as a benchmark. Evidence is graded from the number of independent datasets, the consistency of their direction, the availability of adjusted estimates and geographical breadth. All studies are observational, so every direction is an association and not a causal effect.

**Abbreviations:** ARRO, Association of Residents in Radiation Oncology; ASTRO, American Society for Radiation Oncology; CI, confidence interval; ESTRO, European Society for Radiotherapy and Oncology; NCI, National Cancer Institute; NIH, National Institutes of Health; OR, odds ratio; RANZCR, Royal Australian and New Zealand College of Radiologists; RR, risk ratio; SCAROP, Society of Chairs of Academic Radiation Oncology Programs; URM, under-represented in medicine.

### 3.2 The “leaky pipeline”: Representation across training and academic rank

A consistent finding across all regions is the “leaky pipeline”, the attrition of women between entry and senior rank. In the United States women were 40.0% of 268 medical student members of the American Society for Radiation Oncology but 31.5% of 713 members-in-training (*p* = 0.032) [35]. Student intention surveys and an analysis of 487 letters of recommendation detected no significant gender difference [[39], [40], [41]], which is a failure to detect rather than a demonstration of equivalence; students were, however, more likely to enter the specialty from medical schools with a larger number of female radiation oncology faculty (*r* = 0.481; *p* < 0.001) [36]. Resident representation has held between 27.1% and 34.8% for four decades [[24], [42], [43], [44], [45], [46], [47], [48], [49], [50]], and its growth flattened to 0.4% between 2016 and 2020, the lowest of 16 specialties [51].

In practice the proportion is lower: 1164 of the 4483 radiation oncologists practising in the United States in 2016 were women [29], a figure consistent with the other national workforce counts identified [[8], [52], [53], [54]]. In Canada men outnumbered women at least 1.5:1 among 510 radiation oncologists [55]; women reached 37% (217/582) by 2019, but female trainees fell from 52% to 35% by 2021 (*p* = 0.011) [56]. Women more often accepted academic first posts [[57], [58]], while no significant gender difference was detected in academic career attainment, intention or geographical placement [[59], [60]]; women more often faced spouse-related restrictions on a first post but less often an undesirable location [59].

Faculty representation rose from 10 of 119 in 1970 to 615 of 2115 in 2019, and rose more slowly for women who are under-represented in medicine (*p* < 0.001) [15]; two further cross-sectional counts of academic faculty describe the same distribution [[32], [61]]. Among 993 radiation oncologists at 51 National Cancer Institute-designated centres, 24.6% of men but 15.5% of women were full professors (*p* = 0.001), and 11.8% of the 51 department chairs were women [11]. Four underpowered analyses detected no significant difference in rank or promotion (odds ratio 0.86 for associate or full professor rank among 858 faculty; 95% CI, 0.56–1.32; *p* = 0.48) [[16], [17], [19], [28]].

Outside North America the evidence is thinner. Women were 51.5% of the 75 French residents surveyed in 2008 [12], against approximately 44% of the practising French workforce, a background figure quoted in the 2024 French national survey [13], and held 18.9% of German full professorships in 2024 [10]. In the German analysis of oncological guideline panels, the academic qualifications of panellists differed significantly by gender (*p* < 0.001), 81.3% of the 1676 professors sitting on those panels being men; that figure covers all oncological specialties combined and is not specific to radiation oncology, and within the same study the academic qualifications of the radiation oncology mandate holders did not differ significantly by gender (*p* = 0.35) [22].

Australasian censuses recorded 60% men among 514 radiation oncologists, trainees 48.5% women [[62], [63], [64], [65], [66], [67]]; among 463 European professionals under 40 only radiobiologists were skewed, 80% men [68]. In South Asia 1370 of 4074 radiation and clinical oncologists were women [69], a single qualitative study of 14 senior women in Japan and the United States lacks a denominator [70], and for Latin America only a Mexican census, 99 of 368 certified radiation oncologists being women [38]. No such study was identified for Africa or the Middle East.

### 3.3 Leadership, recognition and visibility

Leadership in specialised societies and editorial boards also shows a marked gap.

In a 2013 national profile of US academic programmes, women were 9% of 76 chairs and 24% of 87 programme directors [71], and 11.7% of chairs against 30.7% of faculty, significantly below expectation [72]; 9 of the 80 chairs (11.3%) at institutions with an accredited residency programme were women (*p* < 0.01) [73]. A survey of 72 chairs, 10 women, found no rank, publication or prior‑leadership difference [19]. Among 4074 South Asian oncologists the female to male ratio was 1: 2, none heading a department in Nepal or Pakistan [69].

Men held 72.7% of 205 seats and 73.5% of leadership roles on four North American committees (both *p* < 0.0001) [18]. Women were 126 of 366 ASTRO guideline panellists [74] and 34.5% of 255 German radiation oncology panellists, significantly above the all-oncology average (*p* < 0.05) [22], but 73.1% of diversity leaders [75] and 70.6% of wellness leaders [76]. Male fellows more often led RANZCR and Trans Tasman bodies [77]. The Brachytherapy Society journal board held fewer women than ASTRO's (18% vs 43%; *p* = 0.02) [78]. Of 1657 repeat reviewers 24.9% were women, with no difference detected in timeliness, quality or acceptance [[79], [80]].

Women were 27% of 725 invited ASTRO speakers [81] and 43.3% of 2973 faculty later, manels falling at ASTRO (*p* < 0.001) but not CARO [82]. They were 29% of 233 visiting professors [83] and 38.9% of grand rounds speakers [84]. Female Educator of the Year recipients rose significantly (*p* < 0.01) [21]; RANZCR prizes showed no winner-applicant difference [85]. Evidence is North American; Asia only a South Asian registry and 14 senior women in Japan and the United States [70], none from France, the United Kingdom, Italy, Spain, the Nordic countries, Latin America, Africa or the Middle East.

### 3.4 Research productivity, authorship and bibliometric standing

Female authorship has risen steadily: first authors in the Red Journal rose from 13.4% to 29.7% by 2012 [43]. Among 420,526 oncology authors, women were less often last than first authors regardless of journal type, including radiation oncology journals [86]; elsewhere only the overall female proportion rose (*p* = 0.005), coinciding with double-blind review [87]. Australasian first- and senior-author proportions were 41% and 32%, both falling in 2021 [88]. Among 168 RANZCR radiation oncology fellows who completed training between 2014 and 2023, the number of first-author publications differed significantly by gender (*p* = 0.04), as did the number of any-author publications (*p* = 0.002), neither accompanied by an effect estimate [27].

None of these analyses covered continental Europe, Asia or Latin America. Acceptance, peer review and abstract-to-publication conversion showed no detectable difference [[89], [90]], though mixed-gender dyads had higher odds of high-impact publication [23].

Crude bibliometric gaps favour men but are attenuated or abolished by stratification for rank. Among 826 faculty, women had a lower mean h-index (6.4 versus 9.4), a difference that all but disappeared when faculty were stratified by rank [25]; among 1031 faculty, men led on m-index, h-index and publication counts (all *p* < 0.05), differences that were largely non-significant after stratification for rank [61]. Male gender correlated with h-index and citation ratio elsewhere [[91], [92]], though only among professors in one analysis [28] and not on the m-index among committee members [18]; career duration was itself a strong correlate (*p* < 0.001) [93], and h-index, not female sex, predicted promotion, an underpowered null [17].

In 607 residents the deficit vanished when the analysis was stratified by position [47], though in 910 graduates female gender predicted fewer publications (*p* = 0.005) [24] and male gender more first-author output [94]. Unadjusted analyses of the 2016 resident class [[95], [96], [97]], of pre-residency output [48] and of survey data [98] did not detect a statistically significant gender difference; all were underpowered. Only the m-index normalises for career length, where the difference was smaller or absent. Surname change at marriage, untraced in every study, fragments women's records, and rapid workforce change leaves women earlier in their careers, understating cumulative output. During COVID-19 one journal saw no overall fall but a significant fall for non-senior female first authors (*p* = 0.02) [26]; ESTRO Twitter interactions fell from 9097 to 2945, and 58% of the accounts involved were male-owned [99].

### 3.5 Clinical research: Principal investigators, funding, compensation and disease-site allocation

Of 340 United States radiation oncology principal investigators in NIH RePORTER, 76% were men [28]; otherwise, differences in rank, funding, grant receipt and awards were largely non-significant [[85], [98]], although the 10 women among 72 responding chairs held greater extramural funding (median $1.89 million versus $0.25 million; *p* = 0.006) [19], and about one in five of 465 Trans Tasman Radiation Oncology Group trial principal investigators were women [100].

In 2016 Open Payments data, 61.4% of practising women (715 of 1164) versus 70.4% of men received an industry payment, with lower median consulting and honoraria payments to women [29], who were 29% of 233 visiting professors and received lower honoraria (*p* = 0.035) [83].

Unadjusted and adjusted estimates diverge: among 247 public-sector faculty (81 women) a 12.6% unadjusted gap fell to 6.4% ($26,458) after adjustment (*p* < 0.01) [30], while among 858 academic radiation oncologists (33.2% women) regression found none (−1.7%; 95% CI, −6.8% to 3.4%; *p* = 0.51) [16]; 58.9% of 296 South Asian women, with no male comparator, reported income similar to male colleagues' [101]. Among 4393 radiation oncologists in 2016 Medicare data, women collected $143,610 less revenue in non-facility-based practice (95% CI -$185,528 to -$101,692; *p* < 0.001) and less in facility-based practice (*p* < 0.001); these measure billing activity, not salary, unadjusted for case mix [31].

Among 1337 academic faculty (30.5% women), women were twice as likely to treat breast (RR 2.01; 95% CI, 1.75–2.50) and gynaecological malignancies (RR 2.06; 95% CI, 1.72–2.79), and men three times more likely to treat genitourinary cancer (RR 0.40; 95% CI, 0.34–0.48; *p* < 0.001 for each), with no difference in number of sites treated (*p* = 0.29) [32]. Breast also predominated among mentorship-programme trainees (50.4%) [102]; 9 of 13 genitourinary and 3 of 31 gynaecological respondents reported gender-related challenges [103]; and the 2018 RANZCR census did not report subspecialisation by gender. Evidence is United States and Australasian, with one South Asian survey and none from Western Europe or Latin America.

### 3.6 Self-reported barriers, workplace environment and parenthood

Resident surveys report gendered environments: among the 203 respondents to a survey of 611 US residents, women more often perceived gender-specific bias in their professional and learning environment (*p* < 0.001) [50]; 27% of women-only respondents reported unwanted sexual advances, no male comparator [49]; male residents more often felt respected (*p* < 0.008) [104]; Canadian trainees (98 of 177) reported more discrimination among women (*p* < 0.001) [33].

Among 144 society members (31.3%), 35.7% reported unwanted advances [105]; ASTRO introductions did not differ by speaker gender (*p* = 0.550), though male introducers used professional address less often (*p* < 0.001) [20]. Mentorship findings are mixed: less satisfaction and more difficulty finding mentors among women [37], but two detected no difference in access [106]; interviews report persistent sexism [70].

On parenthood, 44.2% of 190 respondents reported pregnancy during residency, publications not differing by parental status [42]. Of 126 early career women, 58% delayed pregnancy for the boards, more often reporting infertility (*p* = 0.008) [34]. A Canadian survey found longer first-child leave among women (29 vs 6 weeks, *p* < 0.001), no significant difference detected for parenthood as career-harming (*p* = 0.085) [107]. Paternity leave was examined once (108 of 536 US men): four weeks allowed, two taken [108]. Only 5% of women residents reported no burnout [49], and women perceived more strain (*p* < 0.001) [50].

The French survey is the only national discrimination study in Western Europe: of 225 questionnaires (19.2% response rate, women over-represented), 65% of women said gender had harmed their career, 62% reported inappropriate behaviour or sexual harassment, and 38% of women against 8.5% of men felt a child had greatly affected their career (*p* < 0.001), with no gender difference in career satisfaction [13].

A 35-country survey of 812 professionals found minority-gender respondents scored lowest (47%) [1]; German work identifies inadequate parental-leave substitution, limited part-time pathways and temporary contracts [109]. Australasian surveys describe composition, not discrimination (women less often full time, untested) [65]; part-time work was likewise more common among Canadian women (*p* < 0.01) [55]. A South Asian survey of 296 women, no male comparator, found maternity leave partially denied to 77 of the 176 mothers among them (43.8%) [101]. No study from Latin America was identified.

### 3.7 Interventions that have been evaluated

Several initiatives have reported outcomes, all uncontrolled. The SWRO Mentorship Program's 296 self-selected participants entered academia more often than a reference proportion (82.1% versus 58.3%; *p* < 0.0001) [102]. US residents in a formal mentoring programme reported greater satisfaction (odds ratio 5; *p* < 0.001); women were less likely to be satisfied, without an effect estimate [37]. A mentorship initiative in 58 students detected no difference in output by participation or gender [110].

Departmental initiatives were assessed before and after, without controls: one improved training and education (*p* < 0.001) but not departmental bias (*p* = 0.130) [111]; 34 diversity leaders of 124 US departments described infrastructure rather than effect [75]; one residency programme reported diversity rising 40 percentage points from 1992 to 1995, untested [112], [113]. No controlled evaluation was identified, and no study measured the effect of an initiative on promotion, funding or retention.

### 3.8 Quality of the underlying evidence

Four patterns recur across the 108 included studies.

Gender is more often attributed than reported. Gender was self-reported in 43 studies, from an administrative field in 18, attributed by investigators in 27, and unstated in 20. Attribution misclassifies non-randomly, entangling gender with ethnicity and training country. All categories were binary.

Adjusted estimates and confidence intervals are rare. Sixty-one of the 108 studies report a *p* value, only 17 an adjusted effect estimate and only 10 a confidence interval. Comparisons are often called significant or non-significant with no effect size, so imprecise large effects cannot be distinguished from precise small ones.

Several null results are underpowered rather than reassuring. Crude differences often become non-significant after stratification by academic rank in samples not powered for it: failures to detect, not equivalence, yet cited as parity. Rank is itself an outcome, so adjusting for it removes part of the disparity.

Denominator ambiguity is endemic. Percentages frequently lack denominators, and gender-conditional proportions are misread as whole-sample proportions. Response rates are frequently below 50%, and no identified survey compared responders with non-responders; we therefore state denominators and response rates throughout.

## 4 Discussion

### 4.1 Principal findings, causal interpretation and generalisability

Gender disparities in academic medicine are not unique to radiation oncology but transcend specialty boundaries and national contexts. In radiation oncology research, a persistent “glass ceiling” continues to hinder women's progression into senior academic leadership.

Where more than one study addressed the same domain, the direction of effect was consistent across independent datasets and countries. That consistency is the most robust feature of this evidence and is difficult to account for by individual career preferences alone. The evidence does not, however, establish three things. It does not establish the magnitude of any disparity with useful precision, since only 10 of the 108 studies report a confidence interval. It does not establish that observed differences are attributable to gender rather than to career duration, cohort composition, part-time working, disease-site allocation or institution, since only 17 studies report an adjusted estimate, several adjusting for academic rank, itself one of the outcomes at issue. And it does not establish any mechanism, since no study was designed to test one.

Two consequences should be considered when interpreting this review. First, all reported relationships are associations, not causal effects. The included studies are cross-sectional, registry-based, bibliometric or survey-based, and the present synthesis is narrative; neither the primary studies nor the synthesis supports causal inference. Differences in senior positions, authorship, leadership roles or reported discrimination may reflect gender-related factors but may also be influenced by career duration, cohort, subspecialty, institution, sector or working time, with only 17 studies reporting adjusted estimates. Terms such as “barrier,” “disadvantage” and “leaky pipeline” are therefore descriptive rather than causal. Similarly, temporal changes in representation do not demonstrate an effect of any intervention.

Second, generalisability is limited by substantial geographical imbalance. Of the 108 included studies, 82 (76%) were North American, 10 (9%) Australasian, 8 (7%) multinational, 5 (5%) Western European, 2 (2%) Asian and 1 (1%) Latin American. No radiation-oncology-specific, gender-disaggregated studies were identified from Africa, the Arab countries of the Middle East and North Africa, Eastern Europe, or most of Asia. Evidence from Western Europe was also limited to a small number of German and French reports. Because healthcare systems, career structures, funding mechanisms, workforce composition and the social and legal context for women vary substantially across regions, the magnitude or even presence of reported disparities cannot be assumed elsewhere. The proportions presented therefore describe only the systems studied; whether comparable disparities exist in underrepresented regions remains unknown. Further research in these regions should therefore be prioritised.

### 4.2 Proposed mechanisms: Hypotheses rather than findings

The mechanisms discussed here are hypotheses, none tested within radiation oncology; they generate testable predictions but are not supported by the evidence in Section 3.

The proposed causal chain runs thus: gendered disease-site allocation channels women towards sites that attract less industry funding; reduced funding limits access to collaborative networks and large datasets; restricted access limits senior-authorship output; and reduced output slows bibliometric accumulation and therefore promotion. Two links are documented separately, disease-site distribution and the productivity gap, but no study has tested the chain or shown that allocation mediates the observed differences; testing it would require a longitudinal design with formal mediation analysis.

The “Matthew Effect” of cumulative advantage has been proposed to disadvantage women with smaller start-up packages or less sponsorship, lowering h-indices and grant success. Others invoke the “invisible service tax” of administrative and teaching work [114], lower reported risk-taking, competitiveness and confidence [7], and unconscious bias in promotion [115]; none has been quantified in radiation oncology. The “Matilda effect” describes systematic under-recognition of women's science [116]; a historical analysis documents further examples from the specialty's early history [117].

The studies in Section 3 measure representation, output and reported experience, not mechanism.

### 4.3 The wider academic-medicine context

This section reports general academic medicine and oncology, not radiation oncology; effect sizes observed elsewhere should not be assumed to transfer.

European academia leaks: across the European Union in 2018 women held 46.6% of grade C academic posts but 26.2% of grade A posts [118], and women were 27.7% of the 39,240 principal investigators in oncology clinical trials whose gender could be determined [4].

In France, women held fewer than one in five of 3950 professorships awarded [119]. Elsewhere, women were fewer last authors in Cochrane oncology reviews, and the pandemic, childcare, scarce role models and undervalued teaching weigh more heavily on them; a 2025 French report found 53% prevalence of sexist or sexual harassment [120].

Intersecting identities amplify barriers: Black women are under 0.5% of UK professors, and LGBTQIA+ individuals face harassment and exclusion [121]. Within radiation oncology, the French survey [13] and the Canadian survey [14] are the only primary sources we identified on intersecting disadvantage. Diverse teams have been argued to improve care, education and research innovation [122].

### 4.4 Interventions: Described more often than evaluated

The interventions most frequently recommended here (protected research time, structured mentorship and sponsorship, transparent promotion, substitution during parental leave, viable part-time academic tracks, and equitable representation on guideline bodies) are consistent with the barriers reported and supported by organisational research outside medicine [123]. As Section 3.7 reports, several initiatives have published outcomes within radiation oncology, but every one is uncontrolled and none reports an effect on promotion, funding, retention or academic rank. That literature supports the claim that structured programmes can be delivered and are valued, not that any narrows a disparity.

Several national initiatives have emerged: the Society for Women in Radiation Oncology (SWRO); in Germany, the Women in Radiation Oncology Working Group (FiRO), established in 2024 [124]; and in France, FORce (Femmes Oncologues Radiothérapeutes pour la coopération et l'équité), created in 2025.

This evidence demonstrates feasibility and acceptability, not efficacy. The initiatives use self-selected participants, no concurrent comparator, and short-term outcomes such as participation, satisfaction or academic entry, unadjusted for enrolment characteristics; none assessed promotion, leadership attainment, grant success or retention, and none used a control group or stepped-wedge design. No conclusion can therefore be drawn on whether mentorship, leadership initiatives or institutional policies reduce gender disparities in radiation oncology: these measures are proposed as reasonable, barrier-informed strategies, not proven solutions, and should be implemented in ways that allow evaluation.

Each is well placed to move from description to evaluation by building a pre-specified outcome, a defined comparison group and published results into its activities. A cluster of departments adopting a defined intervention, with contemporaneous departments as comparators and academic rank or grant success as the outcome, would be the most valuable addition to this literature.

### 4.5 The French context and a research agenda

Four things are unknown for French radiation oncology. There is no published, gender-disaggregated analysis of academic rank (PU-PH, MCU-PH, chef de clinique); no French data on gender and research output, covering authorship, principal-investigator roles, PHRC and INCa grant success, HDR attainment or protected research time; and no data on trajectory, the existing survey being a single cross-section that cannot identify at which transition attrition occurs. Its 19.2% response rate, with over-representation of women, means its prevalence estimates are not population estimates.

A national survey conducted with SFRO, SFjRO and FORce should therefore use the complete SFRO or CNOM sampling frame with active follow-up and publish a comparison of responders with non-responders; collect self-reported gender with a non-binary option; link reported experience to verifiable outcomes such as academic rank, HDR status, protected research sessions, grant success and authorship position; pre-specify adjusted analyses accounting for age, seniority, disease site, sector and part-time status; and establish a repeatable baseline, so that the effect of FORce and comparable initiatives can be evaluated prospectively.

### 4.6 Limitations

Four features separate this review from a systematic review. Screening was performed by a single author, so borderline records may be misclassified; the studies retained and the data extracted into Table 1, Table 2 were, however, independently checked against the source reports by a second author. We registered no protocol. We applied no validated risk-of-bias instrument, none existing for bibliometric and workforce-census designs, using instead the domain-based appraisal of Section 2.5. And data were extracted largely from abstracts, so figures given only in a paper's body are absent from our tables.

Embase, Scopus and Web of Science were not searched, for want of institutional access, so reports indexed only there, in regional indexes or unindexed may have been missed. This is our search's most consequential limitation, although Europe PMC yielded 315 records absent from PubMed, six eligible. Retrieval is biased towards English-language indexing, and null analyses may be less likely to be published. We judge this risk moderate rather than negligible: the marginal yield of the second database was low, though grey literature and reports indexed only in regional databases remain outside our search.

The underlying evidence, appraised in Section 3.8, is more constraining: non-comparable structures and rank definitions across countries; cross-sectional, registry-based, bibliometric or survey designs supporting no causal conclusion; binary gender categories; investigator-attributed gender; vulnerable bibliometric measures; inconsistent adjustment; low response rates without responder comparison; underpowered null findings presented as parity; concentration in North America and Western Europe; and unappraised contextual sources in Section 4.3. Two of these limitations govern interpretation: no design included in this review can establish causation, so nothing reported here should be read as an effect of gender; and no evidence at all was identified for Africa, the Middle East, Eastern Europe or most of Asia, so this review describes the academic systems that have been studied and not those that have not.

## 5 Conclusions

Gender disparities in clinical and academic radiation oncology remain evident across multiple countries and career domains, including leadership, research, funding, and authorship. Current evidence is predominantly observational and does not establish the underlying mechanisms or demonstrate the effectiveness of corrective interventions. These are associations, not causal effects of gender, and no programme has been shown in a controlled evaluation to change promotion, leadership or funding. The evidence is also predominantly North American and should not be extrapolated to academic systems that have not been studied. Future studies should use standardised, gender-disaggregated reporting, self-reported gender wherever feasible, and adjusted estimates with confidence intervals. In France, a national survey conducted with SFRO, SFjRO and FORce would provide the baseline the specialty still lacks. Ensuring equitable academic opportunities remains essential for the future of the specialty.

## Patient consent statement

Not applicable. This manuscript is a literature review based exclusively on published literature and publicly available data. It does not include individual patient data, patient images, or any identifiable personal information.

## Consent for publication

Not applicable.

## Ethical approval

Institutional review board approval was not required. This study did not involve human participants and relied exclusively on published literature and publicly available data.

## Declaration of generative AI and AI-assisted technologies in the writing process

During the preparation of this work, the authors used Claude (Anthropic) to assist with English language editing and, where appropriate, to improve the wording and clarity of sentences. After using this tool, the authors reviewed and edited the content as needed and take full responsibility for the content of the publication.

## Funding

This research did not receive any specific grant from funding agencies in the public, commercial, or not-for-profit sectors.

## Declaration of competing interest

The authors declare that they have no known competing financial interests or personal relationships that could have appeared to influence the work reported in this paper.

## Data Availability

All data analysed in this review are available from the cited publications. No new data were generated.

## Appendix A Supplementary data

Supplementary material Full search strategies for PubMed/MEDLINE (searched on 10 August 2026; 749 records) and Europe PMC (searched on 12 August 2026; 880 records), with notes on search syntax.
