# Supplementary material for: Gender disparities in academic radiation oncology research: A narrative review
Source: Clin Transl Radiat Oncol. 2026 Sep 27;62:101298. doi: 10.1016/j.ctro.2026.101298 (PMC13639974; doi:10.1016/j.ctro.2026.101298)
Supplement: Supplementary file 1 [file mmc1.docx]

# Appendix A. Search strategies

## A.1 PubMed/MEDLINE

**Date of search:** 10 August 2026. **Records retrieved:** 749.

("Radiation Oncology"[Mesh] OR "Radiotherapy"[Mesh] OR "radiation oncology"[tiab] OR "radiation oncologist"[tiab] OR radiotherap*[tiab] OR brachytherap*[tiab])
AND ("Sexism"[Mesh] OR "Physicians, Women"[Mesh] OR gender[tiab] OR women[tiab] OR female*[tiab]) AND ("Faculty, Medical"[Mesh] OR "Career Mobility"[Mesh] OR "Authorship"[Mesh] OR "Leadership"[Mesh] OR workforce[tiab] OR academ*[tiab] OR faculty[tiab] OR authorship[tiab] OR professor*[tiab] OR "h-index"[tiab] OR bibliometric*[tiab] OR grant*[tiab] OR leadership[tiab] OR career[tiab])
AND ("2000/01/01"[Date - Publication] : "2026/08/10"[Date - Publication])

## A.2 Europe PMC

**Date of search:** 12 August 2026. **Records retrieved:** 880.

(TITLE_ABS:"radiation oncology" OR TITLE_ABS:"radiation oncologist" OR TITLE_ABS:radiotherap* OR TITLE_ABS:brachytherap*) AND (TITLE_ABS:gender OR TITLE_ABS:women OR TITLE_ABS:female* OR TITLE_ABS:sexism) AND (TITLE_ABS:workforce OR TITLE_ABS:faculty OR TITLE_ABS:academic OR TITLE_ABS:professor* OR TITLE_ABS:authorship OR TITLE_ABS:bibliometric* OR TITLE_ABS:"h-index" OR TITLE_ABS:grant* OR TITLE_ABS:leadership OR TITLE_ABS:career OR TITLE_ABS:resident* OR TITLE_ABS:trainee* OR TITLE_ABS:mentorship OR TITLE_ABS:harassment OR TITLE_ABS:discrimination OR TITLE_ABS:compensation OR TITLE_ABS:salary OR TITLE_ABS:promotion) AND (FIRST_PDATE:[2000-01-01 TO 2026-08-12])

## A.3 Notes on syntax

In the PubMed strategy, [Mesh] denotes a Medical Subject Heading, the controlled indexing vocabulary; [tiab] restricts a term to the title and abstract fields, so that it is not matched in author affiliations or journal names; [Date - Publication] limits the publication date range; and the asterisk is a truncation operator, so that radiotherap* retrieves radiotherapy, radiotherapies and radiotherapeutic.

Two features of PubMed syntax are worth stating, because each fails silently rather than returning an error, and both affected our initial search. PubMed does not expand truncation inside quotation marks, so "radiotherap*"[tiab] returns no records. Nor does PubMed distribute a field tag across a parenthesised group, so (gender OR women)[tiab] is parsed as gender[All Fields] OR women[tiab]. Correcting this second error changed the yield of individual searches by up to an order of magnitude.

In the Europe PMC strategy, TITLE_ABS: restricts a term to the title and abstract. This prefix is required on every term: without it, Europe PMC searches the full text of open-access articles and the same terms return more than 250 000 records.
